# Incidence and Risk Factors of Tuberculosis among Children Receiving Antiretroviral Therapy in Northwest, Ethiopia

**DOI:** 10.3389/ijph.2025.1607892

**Published:** 2025-03-20

**Authors:** Getaneh Endalew, Melkamu Bedimo Beyene, Ayalew Kassie, Gizachew Tadesse Wassie

**Affiliations:** ^1^ Department of Epidemiology and Biostatistics, School of Public Health, College of Medicine and Health Science, Bahir Dar University, Bahir Dar, Ethiopia; ^2^ Department of Nursing, Bahir Dar Health Science College, Bahir Dar, Ethiopia

**Keywords:** ART, child health, WHO clinical staging, incidence, infectious disease

## Abstract

**Objectives:**

Tuberculosis (TB) is a significant global health issue, especially for children living with HIV/AIDS. Hence, the objective of this study was to determine the incidence of TB among children on Anti-retroviral treatment (ART) and its predictors in Northwest Ethiopia.

**Methods:**

A retrospective follow-up study was conducted among 428 children on ART using simple random sampling from patient registries (2011–2020). STATA statistical software was used for data analysis. The Cox regression model was used to explore predictors of TB infection.

**Result:**

The study found that the incidence density of TB was 3.37 cases per 100 person-years. The risk factors for TB incidence among children on ART included a history of contact with active TB cases, missed isoniazid preventive therapy, advanced HIV/AIDS stages according to WHO clinical staging, poor drug adherence, and incomplete vaccination status.

**Conclusion:**

The incidence of TB among children on ART is high, particularly within the first year of enrollment. Children with incomplete vaccination, poor adherence, missed isoniazid prophylaxis, a history of TB contact, and advanced WHO clinical stage are at an increased risk of TB incidence.

## Introduction

Tuberculosis (TB) is a bacterial disease caused by the bacterium *Mycobacterium* tuberculosis. Although it is the most preventable kind of disease in the world, it is nevertheless a significant burden of disease in children [1, 2]. It is the primary cause of death in all HIV-positive individuals, which include children and adolescents [3, 4]. Of the 10 million new cases of tuberculosis, 8.6% involved people living with HIV/AIDS (PLWHA). HIV co-infection and tuberculosis co-infection are terms used to describe the co-infections of the two diseases. A person who has HIV infection and an untreated latent TB infection is much more likely to have TB than a person without HIV infection [5]. Tuberculosis infection can be detected by many techniques, such as culture from biological specimens, acid-fast bacilli microscopy, or bacteriologic confirmation if the TB bacilli are identified by Gene Xpert. Assuming that two of the three following pieces of evidence are satisfied, a structured algorithmic approach can be used to diagnose tuberculosis in young children: The presence of TB-related clinical characteristics, TB contact details, and TB-related alterations on chest radiographs. Furthermore, a child’s radiological picture of a miliary pattern, histopathological results consistent with tuberculosis, or the presence of clinical signs and symptoms could all be used to diagnose TB in young children. Nonetheless, it is still very difficult to diagnose tuberculosis in children living with HIV in underdeveloped countries [1, 6, 7].

Every HIV-infected child should be clinically examined for TB at every healthcare facility visit. The goal of this evaluation should be to identify individuals who are likely to have Tuberculosis and require anti-TB medication, in addition to those who should begin Isoniazid Preventive Therapy (IPT) [8–10].

Healthcare professionals may fail to perform or delay a clinical examination when a patient presents with symptoms suggestive of tuberculosis (TB) [11]. The likelihood of mortality and transmission of infection in the home, community, and medical settings may increase with delayed diagnosis of tuberculosis [12, 13]. Many individuals do not show typical symptoms in the early stages of active TB. Thus, early case detection and screening are advantageous [14–16].

Due to the high prevalence of tuberculosis (TB), a significant amount of work will be required to achieve the Sustainable Development Goals (SDGs) and END-TB plans [17, 18]. Although there has been progress, it is anticipated that the End TB Strategy’s goal of eradicating tuberculosis may not be achieved [19, 20]. Although the goal was to reduce the incidence of tuberculosis by 20% between 2015 and 2020, the global 2020 TB report revealed that tuberculosis decreased by only 9% during this period, or approximately 2% annually. Similarly, only a 14% decrease in mortality was observed between 2015 and 2020, falling short of the 35% target [13, 21, 22].

Childhood TB is often ignored due to its many nonspecific symptoms that overlap with other prevalent pediatric infections. However, young children are more likely to have severe tuberculosis, which has a higher mortality rate [4, 23]. Furthermore, compared to older children and adults, neonates and young children are more prone to experience life-threatening forms of TB disease (disseminated TB, TB meningitis) [18, 24, 25].

To meet the targets of the WHO 2030 “End TB” strategy and the goal of eradicating TB and HIV by 2035, TB programs must collaborate with HIV/AIDS patients to ensure sufficient prophylaxis and ART [22, 26–28].

Only a few previous studies have been conducted, despite the fact that the issue still exists in Ethiopia. The majority of these studies have reported inconsistent median times for developing tuberculosis reports and did not concentrate on survival time to tuberculosis development [29–31]. In particular, no research has been conducted in the current study area. As a result, the purpose of this study was to ascertain the prevalence of tuberculosis among children receiving antiretroviral therapy (ART) and its predictors in Northwest Ethiopia.

## Methods

A retrospective follow-up study design was conducted in Bahir Dar city from patient registries from 1 Jan 2011 to 30 Dec 2020. Data were collected from May 01–30/2021. Bahir Dar city is the administrative capital city of Amhara Regional State and is located 552 km northwest of Addis Ababa, Ethiopia. The city has two public referral Hospitals, one public primary hospital and six health centers. Of these, one referral hospital, one primary hospital, and six health centers provide ART services. According to the Amhara Regional Health Bureau report in 2021, there were 913 children on ART in Bahir Dar city.

### Source and Study Population

The study included children aged 0–15 living with HIV who initiated ART at selected public health facilities between January 1, 2011, and December 30, 2020, and received treatment for ≥3 months. Exclusions applied to patients lacking baseline data (e.g., socio-demographics, TB infection timing post-ART initiation, or incomplete start/exit dates). Whereas patients’ charts with missed baseline information (at least the child’s socio-demographics, unknown time of TB infection after treatment invitation, start date, and end date of follow-up) were excluded.

### Dependent Variable

Time to TB development at any time t (event = 1 and censored = 0).

Independent Variables: Age, sex, maternal serostatus at delivery, place of residence, Caregiver type, parental status, family size, treatment failure, viral load, PMCT, clinical stage, CD4 count, Hgb count/anemic status, opportunistic infection, isoniazid preventive therapy, adherence to ART drugs, cotrimoxazole preventive therapy, date of ART initiation, change in ART regimen related to TB, TB contact history, previous TB treatment history, child vaccination status, body mass index.

### Operational Definitions

Active Tuberculosis: Refers to a patient whose diagnosis of tuberculosis has been bacteriologically confirmed or diagnosed by a clinician’s decision.

Incidence of Tuberculosis: Refers to the diagnosis of new cases of tuberculosis in children on ART using bacteriological examination (smear microscopy, TB culture, and Gene Xpert MTB/RIF assay), imaging techniques (Chest radiograph), and histopathology or biochemical analysis of body parts/fluids”.

CD4 count: This is a test that determines the number of CD4 cells in the blood. A CD4 count of less than 350 is considered below the threshold.

Opportunistic infections: Any of the following diseases: Bacterial pneumonia, oral ulcers, Herpes zoster, Pneumocystis carinii pneumonia (PCP), chronic or acute diarrhea, toxoplasmosis of the central nervous system, and Meningitis caused by streptococcal bacteria in HIV-infected children.

Isoniazid preventive therapy: Chemo-prophylaxis used to reduce the risk of developing TB [18].

Cotrimoxazole preventive therapy: Chemo-prophylaxis given to reduce the risk of opportunistic infections [18].

Vaccination status: Children who receive all appropriate vaccines correlated with their age.

TB contact History: Children receiving HIV/AIDS treatment and had a history of contact with active tuberculosis patients before they develop tuberculosis [13].

Events: The outcome of interest, in this case, is the development of tuberculosis in children receiving ART.

Censored: Children who were either lost to follow-up, dropped out and transferred before developing TB, died due to other causes before the end of follow-up or completed the study period before developing tuberculosis were considered censored.

### Level of ART Adherence

Good Level of ART adherence: Children with a score of ≥95% or <2 missed doses per month or <3 missed doses per 2 months were considered as having a good level of adherence.

Fair level of ART adherence: children with a score of 85%–94% or 3–5 missed doses per 30 doses or 3–9 missed doses per 60 doses.

Poor level of ART adherence: children with a score of less than 85% or >6 missed doses per 30 doses or >9 missed doses per 60 doses.

### Sample Size Determination

The sample size for this study was determined using STATA version 14 with the following assumptions: 95% CI, Power = 85, ratio = 1:1, event among unexposed = 12.87%, AHR = 1.99 (functional status) and a 10% non-response rate. Thus, the final sample size was 428 children.

### Sampling Technique and Sampling Procedure

Two public hospitals, namely Felegehiwot Comprehensive Referral Hospital and Adisalem Primary Hospital, and six public health centers, namely Bahir Dar Health Center, Hane Health Center, Abay Health Center, Dagmawi Minilik Health Center, Tis Abay Health Center, and Shimbit were included. The sample size was then allocated proportionally to the patient load of each healthcare facility. As a sampling frame, a list of children on ART was obtained from each health facility using the patients’ computerized medical record numbers. Study participants were recruited from public health facilities: Felegehiwot Comprehensive Specialized Hospital, Adisalem Primary Hospital, Bahir Dar Health Center, Hane Health Center, Abay Health Center, Dagmawi Minilik Health Center, Tis Abay Health Center, and Shimbit.

### Data Collection Tools and Procedure

The data were collected using a data extraction checklist. The starting time for each study participant began from the date of ART initiation. The total follow-up for this study was 10 years. The event was the incidence of Tuberculosis following the start of ART, with the condition diagnosed using bacteriologic examination (smear microscopy, TB culture, and Gene Xpert MTB/RIF assay), imaging techniques (Chest radiograph), and histopathological or biochemical analysis of body parts/fluids. Before the actual data collection, four nurse data collectors and one BSc nurse supervisor were trained for half a day on the objectives and data collection procedures of the study.

### Data Quality Management

Supervisors and data collectors were trained for half a day. Before data entry, the collected data were reviewed and checked for completeness.

### Data Processing and Analysis Procedure

The data were entered using Epi data version 3.1 and then exported to STATA version 14 for analysis. Descriptive analysis was performed to characterize the proportion of socio-demographic, baseline clinical characteristics and treatment-related variables. The results of the study variables were presented using text, tables and figures. The Kaplan-Meier plot was used to estimate the probability of survival time. The life table was used to estimate TB incidence for each subsequent time interval. The proportional hazards assumption was tested graphically, and the global goodness-of-fit test was tested statistically. A bivariable Cox proportional regression model was constructed and those variables having a p-value <0.25 were included in the multivariable Cox proportional hazards regression model. Thus, variables with a P-value <0.05 with 95% CI were considered significant predictors of time to Tuberculosis development. Furthermore, the Cox-Snell residual plot was used to assess the goodness-of-fit of the Cox proportional hazards regression model.

## Results

### Socio-Demographic Characteristics

Following a review of the records of 428 ART children, 415 were included in the final analysis. Thirteen records with missing baseline information (at least the child’s socio-demographic characteristics, unknown time of TB infection after treatment invitation, start date and end date of follow-up) were excluded. In this study, 53.73% of the subjects were male children and 75.9% of them were urban residents (Table 1).

**TABLE 1:** Baseline socio-demographic characteristics of children on Anti-retroviral treatment in Bahir Dar City, Northwest Ethiopia, 2021 (n = 415).

Variable	Category of variables	Outcome		Frequency	Percent (%)
		Event	Censored		
Sex of child	Male	27	196	223	53.73
	Female	33	159	192	46.27
Age of child in years	<5	27	146	173	41.68
	5–10	24	145	169	40.72
	10–15	9	64	73	17.60
Place of residence of the child	Urban	47	268	315	75.9
	Rural	13	87	100	24.1
Family size	2–4 (normal)	44	272	316	76.14
	≥5 (numerous)	16	83	99	23.86
Parental status	Both alive	18	171	189	45.54
	Mother/father dead	24	131	155	37.35
	Both dead	18	53	71	17.11
Caregiver	Parents	36	299	335	80.72
	Sibling	13	28	41	9.88
	Grandparent	6	20	26	6.27
	Orphan center	5	8	13	3.13

### Baseline Clinical Characteristics

Of the 415 participants, 62.65% did not receive adequate follow-up for the prevention of mother-to-child transmission (PMTCT) during their childhood and 47.23% of the children developed opportunistic infections (Table 2).

**TABLE 2:** Baseline clinical characteristics of children on Anti-retroviral treatment in Bahir Dar City, Northwest Ethiopia, 2021 (n = 415).

Variable (sampled = 415)	Category of variables	Frequency	Percent
PMCT	Yes	155	37.35
	No	260	62.65
OI at baseline rather than TB	Yes	196	47.23
	No	219	52.77
History of past TB Treatment	YES	52	12.53
	No	363	87.47
WHO clinical stage at Baseline	Mild/stages 1 and 2	274	66.02
	advanced/stages 3 and 4	141	33.98
CD4 count at baseline	below threshold	199	47.95
	Above threshold	216	52.05
past OI prophylaxis	Yes	274	66.02
	No	141	33.98
baseline Hemoglobin (gm/dL)	Anemic	85	20.48
	Normal	330	79.52
Stunting	HAZ < −2	205	49.4
	Normal	210	50.6
Thinness	BAZ < −2	156	37.6
	Normal	256	62.4
Viral load status at base line in copy’s	Low viral load	395	95.18
	High viral load	20	4.82

### Follow-Up and Treatment-Related Characteristics

**FIGURE 1:**
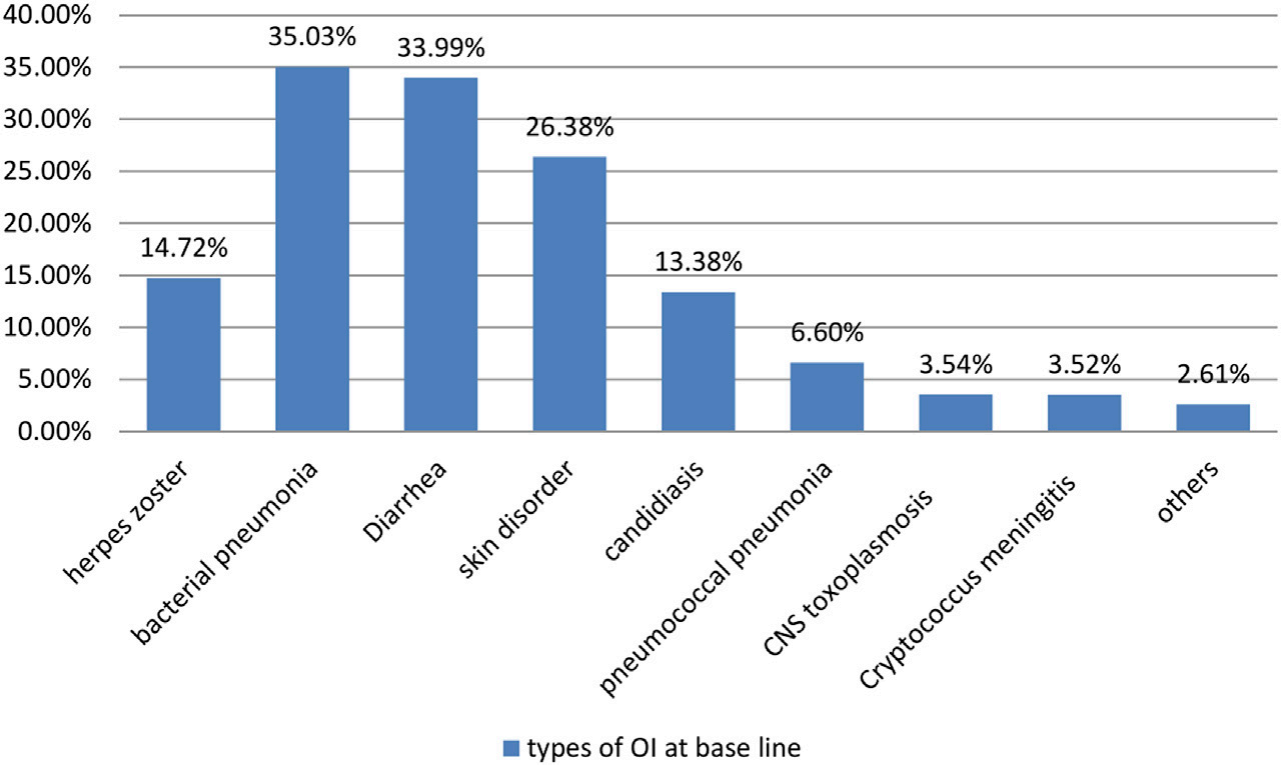
Opportunistic infections at baseline time of children on Anti-retroviral treatment started time at between January 2011 and December 2020 in Bahir Dar City, North West Ethiopia, 2021.

**FIGURE 2:**
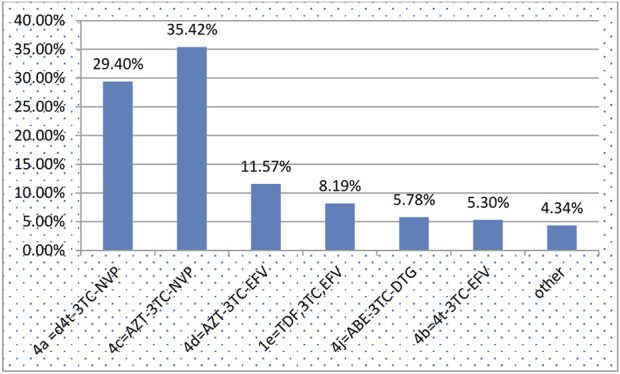
Anti-retroviral treatment regimens for children on Anti-retroviral treatment started time between January 2011 and December 2020 in Bahir Dar City, Northwest Ethiopia, 2021.

During the follow-up period, 67.23% and 15.68% of the participants demonstrated good and fair treatment adherence, respectively (Table 3). The majority of children (35.03%) developed bacterial pneumonia followed by diarrhea (33.99%) (Figure 1). The most common OI developed during follow-up was bacterial pneumonia. The most common regimens prescribed at ART initiation in the cohort were 4c = AZT-3TC-NVP 147 (35.42%), followed by 4a = d4t-3TC-EFV 122 (29.4%) (Figure 2).

**TABLE 3:** Follow-up clinical, immunological, laboratory and treatment-related characteristics of children on antiretroviral therapy between January 2011 and December 2020 in Bahir Dar city, Northwest Ethiopia, 2021.

Variable (sampled = 415)	Category of variables	Frequency	Percent
INH/TB prophylaxis	Taken	283	68.19
	Missed	132	31.81
Cotrimoxazole prophylaxis	Taken	322	77.6
	Missed	93	22.4
Adherence at follow-up	Good	279	67.23
	Fair	65	15.66
	Poor	71	17.11
treatment failure at follow-up	Yes	113	27.23
	No	302	72.77
vaccination status completed	Complete	240	57.83
	Incomplete	175	42.17
TB contact history	Yes	61	14.70
	No	354	85.3
OI development at during follow up	Yes	114	27.47
	No	301	72.53
time from HIV positive confirmation to ART initiation	Timely	201	48.43
	lately	214	51.57

### Time to Tuberculosis Development

A total of 415 children on ART were followed retrospectively in this study between January 2011 and December 2020, for a total of 1782.94 person-years of observation. Thus, 14.46% of individuals developed a new TB infection, resulting in an overall incidence density (ID) of TB development among children of 3.37 (95% CI: (2.612; 4.334) cases per 100 person-years. The median follow-up time was 49 months (IQR = 21.8–73.6), with a minimum of 3 months and a maximum of 120 months. The cumulative probability of tuberculosis-free survival at the end of the study was determined to be 76.5% (95% CI: 69.19%–81.97%).

At the end of the study, 68% of all participants were in follow-up or alive, 20% were transferred to other institutions, 8% were lost to follow-up, and 4% had died. Within 1 year of starting ART, 35% of new TB cases occurred, of which 71.67% were PTB and the remaining 8.33% were EPTB.

The overall Kaplan -Meier survival function showed that there was no median survival time for Tuberculosis development. The majority of TB cases occurred in the first year of ART initiation and decreased during follow-up, continuing steadily in later months of follow-up (Supplementary Figure S1).

### Predictors of Tuberculosis Incidence

In bivariable Cox proportional regression analysis, children’s vaccination status, INH prophylaxis, cotrimoxazole prophylaxis, adherence to drug status, history of treatment failure, a history of contact with active TB patients, hemoglobin status, parental status, caregiver type, Stage of HIV/AIDS according to WHO clinical staging system, CD4 count, history of prophylaxis at baseline, height for age/HAZ and BAZ of children at baseline, viral load and treatment history for TB were variables with a p-value of <0.25 that were candidates for the final model. Then ART adherence status, INH prophylaxis, vaccination status, contact with active TB patients, and Stage of HIV/AIDS according to WHO clinical staging system were variables significantly associated with time to TB development at a p-value <0.05 in the final multivariable Cox proportional regression analysis model.

Children who did not receive Isoniazid preventive therapy were 2.55 times more likely to develop TB than children who received INH (AHR = 2.55: 95% CI, 1.12–5.81). When compared to the fully vaccinated children, HIV-infected children who had received incomplete vaccination had a 2.88-fold (AHR 2.88: 95% CI (1.14–7.28) higher risk of developing TB at any time. The risk of developing TB in children on ART with poor adherence was 3.48 times higher than in children with good adherence (AHR = 3.48; 95% CI: 1.38, 8–80). Furthermore, the risk of developing TB was 3.64 times higher in children on ART who had been exposed to active TB patients (AHR = 3.64; 95% CI (1.73–7.67) compared to children who had not been in contact with active TB patients Finally, Children on ART with advanced baseline WHO clinical stages (3 and 4) had a nearly two-fold higher risk (AHR: 2.18, 95% CI: 1.05–4.53) of developing TB than those with mild stages (1 and 2) (Table 4).

**TABLE 4:** Bi-variable and Multi-variable Cox regression analysis model of predictors of developing tuberculosis among children on anti-retro-viral therapy between January 2011 and December 2020 in Bahir Dar city, North West Ethiopia, 2021.

Variables	Category of variables	Outcome status		CHR [95%CI]	AHR [95%CI]	P-value
		Event	Censored			
Vaccination status	Completed	9	231	1	1	
	Incomplete	51	124	9.52 (4.69–19.35)	2.89 (1.89–7.28)	0.025**
INH prophylaxis	Taken	12	271	1	1	
	Missed	48	84	13.18 (6.99–24.94)	2.55 (1.12–5.81)	0.026**
Adherence	Good	9	270	1	1	
	Fair	13	52	6.76 (7.9–15.83)	2.19 (0.8–5.99)	0.13*
	Poor	18	33	27.46 (13.2119.35)	3.49 (1.38–8.8)	0.008**
Treatment failure	No	12	290	1	1	
	Yes	48	65	9.7 (5.16–18.3)	1.28 (0.56–2.95)	0.56*
Contact history with TB patients	No	18	20	1	1	
	Yes	42	335	16.94 (9.66–29.7)	3.64 (1.73–7.67)	0.001**
baseline Hgb status	Non-anemic	17	313	1	1	
	Anemic	43	42	12.4 (7.07–21.81)	1.15 (0.48–2.75)	0.758*
Parental status	Both alive	18	171	1	1	
	Mother/father dead	24	131	1.73 (0.99–3.19)	1.09 (0.5–2.34)	0.83*
	Both dead	18	53	3.17 (1.65–6.11)	0.81 (0.36–1.8)	0.6
Thinness	Normal	31	228	1	1	
	Thinness (BAZ < -2)	29	127	1.53 (0.92–2.54)	0.95 (0.51–1.78)	0.89*
Stunting	Normal	19	191	1	1	
	(HAZ < −2)	41	164	2.78 (1.61–4.80)	1.56 (0.82–2.93)	0.17*
WHO stage	Mild stage/1 and 2	12	262	1	1	
	Advanced stage/3 and 4	48	93	7.84 (4.16–14.77)	2.18 (1.05–4.53)	0.038**
viral load	Low viral load	52	343	1	1	
	High viral load	8	40	3.08 (1.46–6.48)	1.24 (0.24–3.13)	0.65*
baseline history of TB treatment	No	18	336	1	1	
	Yes	42	19	16.93 (9.66–29.7)	1.35 (0.65–2.82)	0.41*
Cotrimoxazole	Taken	22	300	1	1	
	Missed	38	55	8.18 (4.83–13.86)	1.18 (0.6–2.33)	0.62*
History of OI prophylaxis	Yes	21	147	1	1	
	No	39	102	5.13 (3.01–8.75)	1.81 (0.92–3.56)	0.08*
Caregiver	Parent	36	299	1	1	
	Sibling	13	28	3.62 (1.78–6.34)	1.42 (0.64–3.18)	0.38*
	Grandparent	6	26	2.66 (1.12–6.34)	1.62 (0.57–4.60)	0.36*
	orphan center	5	8	4.8 (1.88–12.29)	2.75 (0.87–8.68)	0.08*
CD4 count	Above threshold	8	208	1	1	
	Below threshold	52	147	7.95 (3.77–16.73)	1.31 (0.53–3.24)	0.55*

1 = Reference category, * = p < 0.25, ** = p < 0.05.

## Discussion

This study revealed that the overall incidence density was 3.37 (95% CI: 2.61; 4.33) cases per 100 PYs of observation. The current result was lower than previous studies conducted in other parts of Ethiopia such as the Beshangul region (9.6 per 100 PYs) [29] and Adama Referral Hospital and Medical College, and Oromia (6.03) per 100 child-years of observation [10]. However, it was higher than those of previous studies conducted in developed nations; the United Kingdom (0.196) per 100 PYs [32], and the United States of America (0.00302) per 100 PYs [33], and Latin America (0.28 per 100 PYs). This disparity may be attributed to the higher incidence of tuberculosis in resource-constrained settings. This suggests that additional efforts are still needed to reduce the incidence of TB, especially among children on ART.

At the end of the follow-up period, the cumulative survival probability of tuberculosis-free children on ART was 76.5% [95% CI: (69.63%; 82.02%)]. This is in line with the results of a study conducted at Gondar University, where the probability of TB-free cumulative survival was 76% [30].

Children with incomplete vaccination status had a higher risk of developing tuberculosis than fully vaccinated children. Previous studies conducted in Gondar [30], Beshangul Gumze, Ethiopia [34] Adama, Ethiopia [10], and Tanzania supported this finding [35]. This may be because BCG vaccination significantly reduces the risk of tuberculosis, particularly in children.

The study also discovered that adherence to ART drugs was a predictor of developing TB. Children with poor ART adherence had a higher risk of developing TB than those with good adherence. This finding was consistent with the results of a study conducted in Debre Markos, Amhara Region, Ethiopia [31]. The possible explanation would be that Poor adherence to ART results in a failure to suppress viral replication, increasing the likelihood of developing HIV mutations that could lead to the development of drug-resistant viral strains. Additionally, poor adherence to ART fails to prevent further viral destruction of the cellular immune system, resulting in a decline in CD4^+^ cell levels and the development of opportunistic infections.

This study demonstrated that advanced baseline WHO clinical stages 3 and 4 of HIV/AIDS were significant predictors of tuberculosis (TB) development in children on ART. This finding was similar with results conducted in UK [32] and Debre Markos, Ethiopia [31]. Deterioration of immunity in advanced WHO clinical stages accelerates the progression of latent TB infection to active TB infection, so children with advanced WHO clinical stages require close monitoring. Advanced HIV disease is associated with immunological deterioration, which leads to the activation of latent TB to active-stage TB. Furthermore, this study found that patients with a history of contact with TB patients were more likely to develop TB than children with no history of contact. Similar findings were reported in Ethiopia’s Beshangule Gumze regional state [29]. This may be due to the latent phase of tuberculosis being activated when immunocompromised children are exposed to active tuberculosis microbes. Finally, children who did not receive Isoniazid/INH prophylaxis had a higher risk of developing TB than their children who did. This finding is also consistent with a previous study conducted in northern Ethiopia [36], and it is supported by a study conducted in Ethiopia’s Adama Oromia regional state [10]. This could be because IPT reduces *mycobacterium* load and slows the progression of latent bacilli to active TB. In Ethiopia, where the prevalence of latent TB infection is high, the guideline for pediatric HIV/AIDS care and treatment recommends IPT for HIV-positive children who have been ruled out of active TB.

### Limitations of the Study

This study did not address some important variables such as the impact of provider training, supplies, and equipment, on child survival due to the retrospective nature of the study. We will recommend to other researchers to conduct a prospective study in this field.

### Conclusions and Recommendations

The incidence of tuberculosis among children on ART was high, particularly in the first year after ART was initiated. Contact history with an active TB patient, lack of Isoniazid preventive therapy, being at an advanced stage of WHO clinical stage, poor ART adherence, and incomplete vaccination status were risk factors for tuberculosis incidence. Hence, the existing TB/HIV prevention and control program should be strengthened to implement all packages that enable the reduction of the high incidence of tuberculosis among children on ART.

## Supplementary Material

SUPPLEMENTARY FIGURE S1 The Kaplan Meier overall survival curve with 95% confidence intervals among children on antiretroviral therapy between January 2011 and December 2020 in Bahir Dar City, Northwest Ethiopia, 2021.
