# Supplementary figures and images for: Incidence and Risk Factors of Tuberculosis among Children Receiving Antiretroviral Therapy in Northwest, Ethiopia
Source: Int J Public Health. 2025 Mar 20;70:1607892. doi: 10.3389/ijph.2025.1607892 (PMC11964899; doi:10.3389/ijph.2025.1607892)

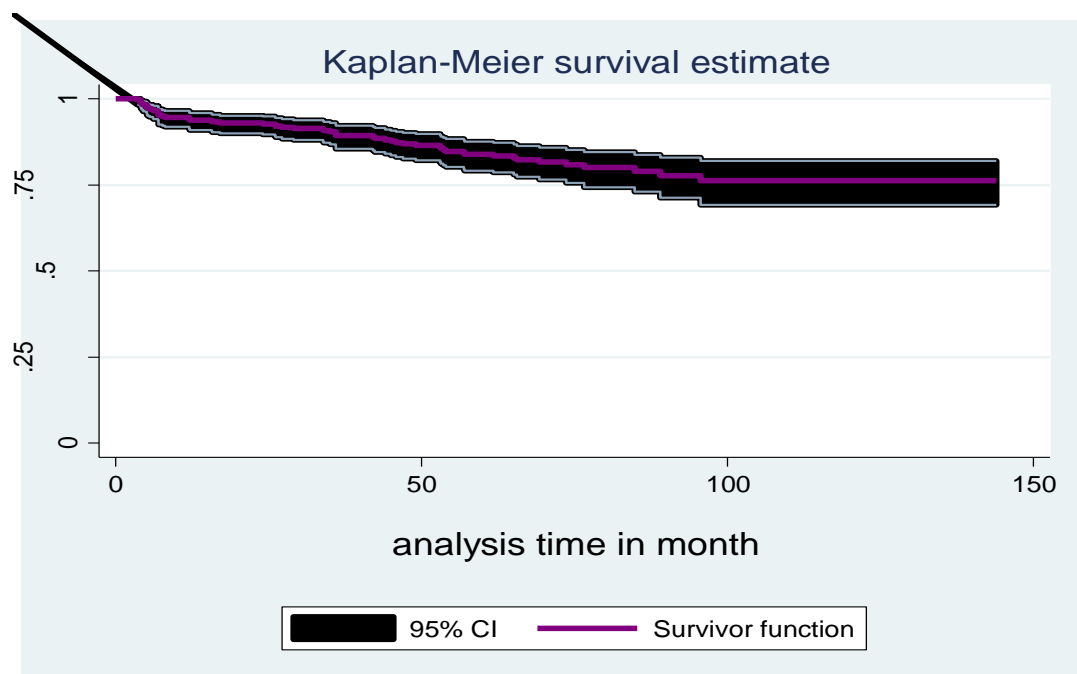

Supplement: Supplementary file 1 [file DataSheet1.PDF]
